# “Old Wine in a New Bottle”. Depression and Romantic Relationships in Italian Emerging Adulthood: The Moderating Effect of Gender

**DOI:** 10.3390/ijerph17114121

**Published:** 2020-06-09

**Authors:** Alessandra Fermani, Ramona Bongelli, Carla Canestrari, Morena Muzi, Ilaria Riccioni, Roberto Burro

**Affiliations:** 1 Department of Education, Cultural Heritage and Tourism, University of Macerata, 62100 Macerata, Italy; carla.canestrari@unimc.it (C.C.); morena.muzi@unimc.it (M.M.); ilaria.riccioni@unimc.it (I.R.); 2 Department of Political Science, Communication, International Relations, University of Macerata, 62100 Macerata, Italy; ramona.bongelli@unimc.it; 3 Department Human Science, University of Verona, 37129 Verona, Italy; roberto.burro@univr.it

**Keywords:** gender, emerging adults, depression, romantic styles, multi–group structural equation model, violence

## Abstract

Intimate partner violence is an important social issue all over the world, and human sciences, in particular, are working to reduce it. Despite this, the topic is a little recognized phenomenon. Understanding the origins and the variables that have an impact on manic-style romantic relationships, as defined by John Alan Lee, is of primary importance, in particular in Italy where the data reveal alarming statistics. Most studies have not controlled for earlier depressive symptoms as a cause of successive depression or as an antecedent of romantic styles. In our study, we investigate the association between depression and romantic style, trying to test the moderating role of the gender variable in 283 Italian emerging adults (139 women and 144 men). In order to achieve this aim, we performed a multigroup structural equation model analysis. The hypothesis that gender moderates the relationship between depression and romantic styles is still yet to be confirmed. Men with high levels of depression do not seem to be able to establish relationships based on commitment, as required by the eros style. Women with high levels of depression are more frequently involved in possessive and demanding relationships or in pragmatic ones, confirming their need for dependence.

## 1. Introduction

Knowing how emerging adults live their early romantic relationships and which are the variables implicated in the choice of romantic styles could be very important for preventing violence against women. Major changes in emerging adults’ romantic relationships bring about the reflection on what makes a relationship long lasting, and which factors contribute most to avoiding aggressive behavior in a couple. While research has shown the tendency to consider sentimental relationships in terms of commitment, the reality in Italy highlights that fidelity and patience are absent in the representations of love styles which are projected in the present. Emerging adults have a strong desire to affirm themselves and their interests but no partners seek this individualistic affirmation in a couple [1].

Specifically, women are more prone to be involved in emotional ties, sometimes victimizing ones, and are more characterized by their fear of being abandoned and rejected by their partner [2]. The victims generally are more depressed and anxious [3,4]. Males do not tend remain freer from social conditioning. Even males often suffer from anxiety and depression due to the pressure of a culture that would like them to be strong, experienced, and reliable in any situation [5,6]. Recent Italian research has shown significant evidence of a tendency for women to treat their bodies as sexual objects. The same orientation exists in the Italian males: virility, male domination, and the idea of reputation “to defend” are the young’s tacit rules [1].

In Italy, the number of women who have suffered from at least one form of physical or sexual violence in the last 5 years amounts to 4,353,000, i.e., 11.3% of women aged 16 to 70. Psychological violence is more widespread among younger women (35% compared to an average of 26.5%) [7]. Thus, in a country such as Italy, where the data reveal alarming statistics about violence, understanding the variables that have an impact on manic-romantic relationships becomes of primary importance.

It is fundamental to expand previous research to include explicit attention to potential gender differences between men and women, who both could take on pathological romantic styles or become victims of violence. For this reason, in order to analyze the connection between romantic styles and violence, in our study, we investigate the association between depression and romantic style, trying to test the moderating role of the gender variable in emerging adults, i.e., in a phase very important for identity explorations and achievement [8]. Depression is important for identifying possible antecedents of aggression and victimization in romantic relationships, considering gender differences [9,10].

Within the literature, physical or psychological aggression are associated with mental health symptoms [11]. In general, the literature describes these connections in relation to female or male victims, but depression has not been presented as a factor able to affect the choice of a romantic style. As mentioned above, most studies have not controlled earlier depressive symptoms as a cause of successive depression. For example, the depression that normally follows the traumas of a violent relationship could be greater if there was already a depressive condition prior to the trauma. Could it be “old wine in a new bottle”?

Much more research is needed on this topic. The psychosocial literature itself recommends further investigations into these aspects in different contexts [12,13].

## 2. Violence against Women in the Italian Data

The Italian context provides the framework for understanding the importance of studies identifying some variables that predict differences in romantic styles (especially violent and manic ones), considering that the relationships of the variables themselves could differ between men and women. The main results of the research highlight that differences in behaviors may depend on the country of origin [14,15] or on the gender of participants [16]. In Italy, violence against women is an important problem since one woman out of three is a victim of physical or sexual violence. If we consider psychological violence, then the data are even more dramatic.

Data from the Italian National Institute of Statistics (ISTAT) show [5] that 31.5% of Italian women from 16 to 70 years old have been victims of sexual or physical violence during their lifetime; 62% of these behaviors have been perpetrated by the ex-partner or by the current partner of the victims. Among Italian women, 13.6% (2,800,000) endured sexual or physical violence from their partners or ex-partners. In particular, 18.9% (2,044,000) of women suffered violence from their former partners and 5.2% (855,000) from current partners. Many women who lived with violent partners in the past left them precisely because of the violence suffered (68.6%). More specifically, the partner’s violence was the main cause for ending the relationship for 41.7% of women, and for 26.8% it was an important element of this decision.

Beyond relationships, 24.7% of Italian women have suffered from at least one episode of physical or sexual violence from non-partner men: 13.2% from strangers, and 13% from known people (6.3% from acquaintances, 3% from friends, 2.6% from relatives, and 2.5% from work colleagues).

The ISTAT report showed that women face threats (12.3%), are pushed or jerked (11.5%), and are kicked, slapped, punched and bitten (7.3%). In a lower percentage they are hit with objects that can hurt (6.1%). More severe forms such as attempted strangulation, suffocation, burning, and the threat or use of weapons appear to be less frequent. Among women who have suffered sexual violence, the most common forms are physical harassment (for example, being touched, kissed or embraced against their will) (15.6%), unwanted relationships experienced as violence (4.7%), attempted rapes (3.5%), and rapes (3%). In 62.7% of cases, the rapes were committed by partners, in 3.6% of cases by relatives, and in 9.4% of cases by friends. Physical violence (such as slapping, kicking, punching, and biting) is also largely the work of partners or ex-partners. Furthermore, young women with higher educational levels are at risk of violence from non-partners or ex partners.

Psychological violence is more widespread among younger women (35% for 16–24-year-olds compared to an average of 26.5% for older women). Italian women, especially those with medium-to-high social standing, struggle to admit violence for reasons of shame.

In 2017 ISTAT created a survey specifically dedicated to the detection of stereotypes about gender roles and, for the first time, to opinions on the acceptability of violence, its spread, and its causes, as well as stereotypes regarding sexual violence.

Telefono Rosa (www.telefonorosa.it), founded in 1988, is an Italian association dealing with intervention and prevention of gender-based violence (including legal and economic assistance, psychological counseling, hospitality in cases of sexual violence or similar acts, information in stalking events, etc.). The last accessible data from “Telefono Rosa Torino” show that 688 women were helped in 2015 and 788 in 2019. As one might expect, an increase in the severity of the violent episodes is associated with a slight increase in the number of victims. In Italy, in 2017, physical violence increased from 62% to 77%, verbal and psychological violence grew from 81% to 87%, and stalking episodes increased from 15% to 36%. The number of women who have been killed in 2018 was 142 [10]. Moreover, online aid requests increased from 2467 (in 2015) to 3018 (in 2016), and 85% of violent events were committed by an intimate partner [17]. Men who commit violence (50%) often go back to being mistreated. Although many international research projects have been carried out on this topic [18,19,20], Rollè et al. [10] affirmed that, in the Italian literature, there is a gap regarding studies about emerging adults’ attitudes.

A social climate of tolerance about violent episodes can have a negative effect on women’s reactions to their own victimization, by deterring them from seeking help or disclosing the violence. Many studies [10] reveal that women themselves seem to show a higher disposition to blame the victim.

In Italy, based on the results of a Telefono Rosa survey [17], the most common stereotypes about gender roles are: “for men, more than for women, it is very important to be successful at work” (32.5%), “men are less suited to deal with household chores” (31.5%) and “it is a man who has to provide for the economic needs of the family” (27.9%). ISTAT [5] found that 58.8% of the population (aged 18–74), without particular differences between men and women, agrees with these stereotypes, and this is more common with increasing age (65.7% of 60–74 year-olds and 45.3% of young people) and among the less educated. Stereotypes are more frequent in the south (67.8%), and less common in the northeast (52.6%).

On the issue of violence in couples, 7.4% of people believe it is always acceptable, or in some circumstances acceptable, that “a boy slaps his girlfriend because she flirted with another man”, and 6.2% find it acceptable that a couple gives one slap every now and then. Compared to the control, 17.7% believe it is always acceptable, or in some circumstances acceptable, that a man habitually controls the mobile phone use or activity on the social networks of his wife or partner. According to gender paradigms, men seem to be more dominant than women and more inclined to justify violent acts following their cultural gender stereotypes. When asked why some men are violent with their partners, 77.7% of respondents answered that women are considered property objects (84.9% of women and 70.4% of men), 75.5% answered because they are drug or alcohol consumers, and another 75% said it was due to men’s need to feel superior to their partner or wife. The difficulty of managing anger in some men was indicated by 70.6% of respondents.

According to 63.7% of the population, violent experiences suffered in the family during childhood are the cause of violent acts, and 62.6% believes that some men are violent because they cannot stand female emancipation. The claimed association is also high, but less so, between violence and religious reasons (33.8%).

To a woman who has suffered violence from her partner, 64.5% of the population would advise to report him and 33.2% to leave him. A total of 20.4% of the population would refer women to anti-violence centers (25.6% of women versus 15.0% of men) and 18.2% would advise them to turn to other services or professionals (psychologists, lawyers, etc.). Only 2% would suggest calling the Telefono Rosa.

The prejudiced belief that women are responsible for the sexual violence they suffer persists to the degree that even 39.3% of the population believes that a woman is able to avoid sexual intercourse if she really wants to, and 23.9% think that women can cause sexual violence through their way of dressing. Furthermore, 15.1% of the population is of the opinion that a woman who suffers from sexual violence when drunk or under the influence of drugs is at least partially responsible. For 10.3% of the population, accusations of sexual violence are often false (claimed more by men, 12.7%, than women, 7.9%). For 7.2%, when faced with a sexual proposal, “women often say no but actually mean yes”. For 6.2%, “respectable women” are not raped. Only 1.9% believe that it is not about violence if a man forces his wife or partner to have sex against their will.

## 3. Gender Differences in the Impact of Depression on Love Relationships

Depression and other mental health symptoms are mainly studied in this context as consequences of violent acts or manic love [11]. Previous studies about the relationships between romantic relationships and depressive symptoms have largely used adult or adolescent populations and focused on positive or negative relationship outcomes on mental health [21,22,23]. Obviously, depression can also be a primary symptom that precedes violent acts and functions as a risk factor. In other words, it is not only an outcome but can be also an antecedent. Furthermore, Santona et al. [24] showed that high levels of emotional responsiveness can also predict aggressive behaviors during adulthood. Marriage and/or cohabitation, and more in general all stable romantic unions, are linked to better physical and mental health for both children, young people, and adults.

The conditions of Italian young people in the transition to adulthood and the time frame that characterizes this transition are protracted over time. Their delays in scholastic training slow down access to the labor market and the creation of stable sentimental situations, their commitment is reduced, they tend to avoid responsibility, and believe that they are the ones who decide for their lives. The instability in living conditions can cause malaise, anxiety about the future, and depression [25].

Lee [26] proposed an interesting typology of love styles and identified three primary types: eros (romantic, passionate love), storge (friendship love), and ludus (game-playing love). Pragma (logical, “shopping list” love), mania (possessive, dependent love), and agape (all-giving, selfless love) are three secondary styles as a mix of the primary ones. Mania is a combination of eros and ludus, pragma of ludus and storge, and agape of eros and storge. These secondary styles have very different properties than their constituent styles. Hence, the Lee love styles are logically related, but each style has independent qualitative properties. In synthesis, there is not only one type of love but rather many different styles. Consistently with Lee [26], Hendrick and Hendrick [27] believed that it is possible, for example, that the same individual can be simultaneously pragmatic in a relationship with a partner and storgic with another one.

This possibility implies that the cause of a love style lies in the nature of the relationship with another person, and so specific socialization practices affect the development of the conceptual love matrix. It is possible that some styles are more susceptible to change through experience than other styles and the six factors may develop during life span. This research shows interesting gender differences for several of the love styles. Women were more storgic (young women are more inclined to consider friendship as a determinant component in love), pragmatic, and manic in love attitudes than men [2,27]. Psychosocial literature explains that women traditionally have been educated to marry both a love partner and a potential provider. Due to this state of dependence on men, women are more pragmatic than men [1,2]. Furthermore, socialized dependency could cause more manic attitudes in females.

Men were more ludic than women, perhaps because they are permissive in their sexual attitudes. According to Hendrick and Hendrick [2,27], historically, women have been more conservative in their sexual attitudes, considering sex as an important act to take care with.

Females in a lot of research show more depression symptoms in general than males [28]. Attili [2] discussed Lee’s love styles theory, trying to understand the antecedents of romantic styles. For example, those who have a secure attachment and live a state of wellbeing are not anxious and accept the inevitable transformations of the couple by adopting love styles such as eros and agape. When the relationship goes on to the end, these persons have to face the pain of loss and the grief of abandonment and separation. Sadness and despair are managed, and usually in about a year the individual is ready to be engaged in new romantic experiences.

It is unclear whether gender differences exist in romantic wellbeing or in the use of romantic relational aggression. Research supports that women are more likely than men to use relational aggression during childhood, but gender differences are more intricate in adolescence and emerging adulthood [29]. Nonetheless, gender differences have not been adequately studied because all-women samples are the norm in research on depressive symptoms and on romantic relationships [22]. Linder, Crick and Collins [30] hypothesized in their research that romantic victimization and relational aggression would be negatively correlated with positive relationship qualities, such as trust, and that they would strengthen negative relationship qualities such as jealousy. Shorey et al. [11] found that increased proneness to shame increases one’s risk of experiencing symptoms of anxiety more strongly for men than women. Male victims of psychological and physical victimization showed, irrespective of proneness to shame, higher levels of anxious symptoms compared to female victims. According to the aforementioned authors, one possible interpretation of these findings can be gleaned from the socialization processes that traditionally teach men to maintain control in relationships, be assertive and not vulnerable, and to avoid emotions. The masculine role norms may create significant gender role stress and conflict, as well as anxiety and depression. Furthermore, many studies revealed that gender role conflict is often associated with shame: with a reduced sense of masculinity and greater proneness to shame, it is possible that men may experience greater anxiety and fall into depression. In other words, depression can be due to the fact that they may feel that they are not living up to the social norms of masculinity.

According to stress and coping models, which assume that adolescent romantic relationships are inherently emotional and involve challenges such as fear of rejection, people with more support from trusted others would be at a lower risk for depressive symptoms when facing challenging romantic situations. On the contrary, people with more depressive symptoms will report more negative experiences of romantic stress [31]. Furthermore, the association between romance and depressive symptoms is stronger for young women. For example, Anderson, Salk and Hyde’s results [22] suggest that, specifically, a mother’s support may be effective in reducing the influence of romantic stress on depressive symptoms for adolescents.

We think that the identification in emerging adults of the antecedents of romantic relationships suggests that gender and the status of depression may play an important role in the development of romantic relational aggression and victimization.

## 4. The Study

Arnett [32] explained that the expression “young adults” may be incorrect because it conveys the idea that adulthood has been achieved. On the contrary, most people aged 20–29 show that they are in a transitional phase where adulthood is close but not yet reached. Arnett [8] distinguished three phases: adolescence (10–18 years), emerging adulthood (19–29 years), and adult age (after 30 years). According to Arnett’s definition, the participants of this study were emerging adults, with their age ranging from 18 to 29 years. This research has considered emerging adults as a privileged area because most of them are not in their first romantic experiences and are in the transitional phase that leads to the formation of a new family.

In Italy, as mentioned above, psychologists have discussed the “delay syndrome”. The symptoms of this delay are: end of the training processes (not before the age of 27); postponement of the entry into the labor market; high age at which the parents’ home is left to achieve housing independence; postponement of the beginning of a stable life as a couple (often not before the age of 35); delay in the transition to parenting (on average, when the woman is 31 years old) [25].

## 5. Aims and Hypotheses

The main objectives of this study were:

(1) To verify whether depression has an impact on the development of healthy or unhealthy relationships. In the light of the previous literature, depression is important for identifying possible antecedents of aggression and victimization in romantic relationships, considering gender differences [9,10] because physical or psychological aggression is associated with mental health symptoms [11]. The depression that can accompany the traumas of a violent relationship could then be greater if there was already a depressive condition prior to the relationship and it could cause violent behaviors.

(2) To evaluate whether a gender variable moderates the relationship between depression and romantic styles. That is, if different relationships between these constructs are possible in emerging adulthood as a consequence of gender. Specifically, we wanted to see if gender is a moderating variable in choosing a romantic style, in particular in the age of key identity explorations and achievement [8].

In order to achieve these objectives, we verified the following main hypotheses: (1) depression has a negative impact on violence and pathological love [6], especially in men, because men are much more likely to be perpetrators of violence than women [5]; (2) gender creates different relationships between constructs, and moderates these relationships, creating two different relational models.

## 6. Method

### 6.1. Participants

The sample consisted of 283 Italian emerging adults (139 women and 144 men), according to the definition of emerging adulthood provided by Arnett, Žukauskienė and Sugimura [33]. Participants ranged from 19 to 29 years of age (M = 24.4; SD = 2.3). Of the participants, 57.7% were students, 34% workers, and 8.2% never worked. Most respondents were single and had never been married (139 men and 130 women). However, 3% claimed to cohabit with someone at the present or to have cohabited with someone in the past, and 86% did not have children. A final background question attempted to measure their current romantic relationships: 65.9% answered with regard to their current partner and 25.9% answered with regard to their most recent partner. Among the respondents, 8.2% had never been in love. Women and men did not differ on this question (χ2 (2) = 0.23, ns).

### 6.2. Procedure

The study used a convenience sampling and a snowball or chain-referral sampling method for the selection of participants [34].

Participants completed the self-report measure, the Children’s Depression Inventory (CDI), the Love Attitudes Scale (LAS), and demographic information. The total time required to complete the questionnaires was approximately 35 minutes.

Participants were given the option of completing the questionnaires during a lesson in social psychology at the Department of Education, Cultural Heritage and Tourism at the University of Macerata Italy, under the supervision of research assistants. All participants were informed that their participation was anonymous. Prior to initiating the study, we obtained permission from the University, and participants were provided with written information about the research aims and asked for their consent for their participation. Approximately 99% of the approached participants chose to participate and completed the questionnaires during the lesson.

For the data analysis, we used the Statistical Package for the Social Sciences (SPSS, version 22, running on Windows—IBM Corporation, Armonk, NY, USA) and R software (R Foundation for Statistical Computing, Vienna, Austria) environment for statistical computing and graphics [35].

### 6.3. Measures

An adaptation of the Children’s Depression Inventory (CDI [36]) was used to assess depressive symptoms. The CDI is a self-report questionnaire aimed at screening sub-clinical depressive symptoms. The CDI has been successfully used in several studies with adolescents or with emerging adults [37]. The Beck’s Depression Inventory (BDI) [38] for adults was used as a model for the development of the Children’s Depression Inventory, which was appropriate for psychiatric and non-psychiatric populations. The CDI items are similar to the BDI items and grouped into five factor areas, including ‘Negative Mood’, ‘Interpersonal Problems’, ‘Ineffectiveness’, ‘Anhedonia’, and ‘Negative Self Esteem’. In particular, for this study it was important to use a scale also able to measure academic performance decrement or academic work difficulty (not present in BDI), sadness, pessimistic worrying, self-blame, crying, irritability, reduced social interest, indecisiveness, negative body image, sleep disturbance, fatigue, reduced appetite, somatic concerns, and loneliness. Since our sample was made up of participants transitioning from adolescence to adulthood, we preferred the CDI over the BDI, consistent with the results of Crocetti et al. [25,39] who used CDI for young people up to 18 years. Moreover, in Italy psychologists underline that the “delay syndrome” affects Italian young people, which delays their transition to adulthood [25]. We only changed the term “school” to the word “university” (items 15 and 21).

Moreover, the BDI suffers from some limitations [40], while the CDI items are clear, detailed, and easy to understand. The time it generally takes for an individual to complete the CDI is about 10 minutes. The CDI consists of 27 items, scored on a three-point scale: 1 (false), 2 (a bit true), and 3 (very true). A sample item is: “I am sad all the time”. Cronbach’s alpha was 0.91.

We also employed the Love attitude scale LAS [24], a 42-item questionnaire designed to measure attitudes towards love.

The questionnaires were translated from English to Italian by a bilingual psychologist. The measure was translated from English to Italian by a bilingual psychologist and back-translated from Italian to English by a second bilingual psychologist. The two English versions were then compared, then the two translators discussed and resolved any discrepancies between the original and the back-translated English versions. The final English version was then translated into Italian by both psychologists.

We conducted a pilot study. Based on feedback from the participants of the pilot study, slight wording adjustments were made so that all items were adequate for an Italian-speaking sample.

The questionnaire combines attitudes towards one’s current, recent, or hypothetical partner with attitudes about love and participants answer questions with their current, recent, or hypothetical partner in mind. If the respondent does not currently have a partner, he or she should answer keeping their most recent partner in mind. If the participants have never been in love, the instructions state that they should provide whatever answer they believe would be true. Participants respond to each item using a 5-point scale, ranging from 1 (strongly agree) to 5 (strongly disagree). The scale is split into six subscales of seven items each, and each subscale represents a different love style: eros (passionate love; e.g., “My lover and I have the right physical ‘chemistry’ between us.”); ludus (game-playing love; e.g., “I enjoy playing the ‘game of love’ with a number of different partners”); storge (friendship love; e.g., “Love is really a deep friendship, not a mysterious, mystical emotion”); pragma (practical love; e.g., “It is best to love someone with a similar background”); mania (possessive, dependent love; e.g., “When my love affairs break up, I get so depressed that I have even thought of suicide”); and agape (altruistic love; e.g., “I would rather suffer myself than let my lover suffer”).

Hendrick and Hendrick’s [27] descriptions of the six factors are excerpted below.

“Eros: Strong physical preferences, early attraction, and intensity of emotion are attributes of erotic love, along with strong commitment to the lover. […].

Ludus: Love as an interaction game to be played out with diverse partners appears to be the main attribute of Ludus types. Deception of the lover is acceptable within proper role limits. There is not great depth of feeling; indeed, the ludic lover is wary of emotional intensity from others. Ludic love has a manipulative quality to it. This aspect results in apparent lower social desirability. It is important to note, however, that there are ludic aspects to many, if not most, love relationships [...].

1. Storge: This style reflects an inclination to merge love and friendship. There is no fire in storgic love; it is solid, down-to earth, and presumably enduring [...].
2. Pragma: Rational calculation with a focus on desired attributes of the lover is central to pragmatic love. In fact, “love planning” might be an apt description [...].
3. Mania: Reading the items suggests that Mania is “symptom love”, based on uncertainty of self and the lover. It may be most characteristic of adolescents, but examples of older manic lovers frequently occur [...].
4. Agape: Lee did not find this style manifested fully in actual human beings. However, the factor results suggest that it is a viable style” [27] (pp.400–401).

The level of Cronbach’s alpha and the percentage of total variance accounted for by each of the factors were, respectively: eros 0.78, 22.7; ludus 0.70, 22.6; storge 0.72, 29.2; pragma 0.77, 31.1; mania 0.70, 24.1; agape 0.73, 24.3.

### 6.4. Ethical Approval

This research was conducted by respecting the APA ETHICAL PRINCIPLES OF PSYCHOLOGISTS AND CODE OF CONDUCT (https://www.apa.org/ethics/code/ethics-code-2017.pdf) and the rules of the Declaration of Helsinki of 1975 (https://www.wma.net/what-we-do/medical-ethics/declaration-of-helsinki/), revised in 2013. As per point 23 of the declaration, this study was approved by the institutional ethic committee (project identification code 0010407- 10-09-2019 minutes of the PhD meeting curriculum in Psychology, Communication and Social Sciences, University of Macerata.

## 7. Analysis and Results

As stated above, the main aim of this study was to examine whether the gender variable (1 males and 2 females) moderates the relationships among attachment (mother and father) and depression on six romantic styles. In order to achieve this aim, we performed a multigroup structural equation model analysis [41,42].

Many criteria have to be considered when evaluating model fit and the first is the chi-square (χ2). A model fits the data well when χ2 is not significant (*p* ≥ 0.05). This statistic is sensitive to sample size so this study followed Schermelleh-Engel et al.’s [43] suggestions, which consider a χ2/df ratio lower than 3 to be adequate. The good fit of the model was valued with the comparative fit index (CFI), Tucker–Lewis Index (TLI), standardized root mean square residual (SRMR), adjusted goodness of fit index (AGFI), and root mean square error of approximation (RMSEA) [44]. A CFI of 0.95 or above indicates a good fit, and a value below 0.90 indicates a poor fit. A normal fit index (NFI) of 0.95 indicates the model of interest improves the fit by 95% relative to the null model. Non-normed fit index (NNFI), preferable for smaller samples, is called also the Tucker–Lewis index (TLI). The model is considered a good fit if the RMSEA index is less than or equal to 0.05. Values between 0.05 and 0.08 are adequate because they suggest a reasonable error of approximation, and if the index is greater than or equal to 0.10, the model is considered a poor fit.

In this study the significance of the standardized path coefficients was determined by comparing the (absolute) t-ratio to a critical t value of 1.96 (*p* ≤ 0.05).

The square root is the difference between the residuals of the sample covariance matrix and the hypothesized model. In this research the items have different range (i.e., in this study some items are 1–3, others 1–5) so it is better to use SRMR (<0.08). Additionally, AGFI values equal or greater than 0.90 indicate a good fit.

The overall fit of the models was determined through a combination of results from the fit indexes, the significance of the standardized path coefficients, and the significance of the indirect effect.

According to Jöreskog and Sörbom [45], to determine the influence of the gender variable the path analysis model was fitted separately to the two datasets. A unique and identical model across genders would indicate that we should assess whether the factor loadings of the model were invariant across the two groups. Otherwise, we should theoretically analyze the differences between the two models [44,46]. In order to check if the model was invariant across the gender factor, we performed an invariance analysis. Invariance analysis is necessary in the validation of a model [44,47]. “Testing measurement invariance becomes an interesting and indispensable issue within the translation process” [47] (*p*. 82). Testing measurement invariance is essential to checking whether results are attributable to group differences or measurement issues [47]. Thus, it is relevant to investigate the invariance across the gender factor in our case.

Before conducting the multigroup structural equation model, we checked the adequacy of the sample size. According to Kline [44], the minimum ratio between numbers of observations and numbers of free parameters should be no smaller than 5:1. In our case it was equal to 5.05 (283 subjects/56 free parameters).

Table 1 and Table 2 show the regression analyses of the LAS on the CDI as predictors in men and women.

The results confirm the hypothesis that gender moderates the relationship between depression and romantic styles. In men, depression had a negative impact on pathological love (mania, ludus) and on storge and pragma. High depression scores were associated with low eros values. In women, high depression scores were associated with high pragma and mania values.

All of these paths were significant (*p* < 0.05), and the model fits the data reasonably well as indicated by multiple indicators of fit: test statistic 10.298, degree of freedom= 8, *p*-value (χ^2^) = 0.245, χ^2^/df ratio = 1.287, CFI = 0.991, TLI= 0.952, RMSEA = 0.046, SRMR = 0.037, and AGFI = 0.997. We performed multigroup structural equation model by testing separate models, including (1) the configural invariance model, allowing all of the parameters to be freely estimated; (2) the metric invariance model, requiring invariant factor loadings; and (3) the scalar invariance model, additionally requiring invariant intercepts.

Invariance requires a change in CFI no higher than 0.010, a change in RMSEA no higher than 0.015 and a change in SRMR no higher than 0.30 for testing metric invariance and no higher than 0.010 for testing scalar invariance [48]. We considered the change in CFI to be the main criterion as suggested by Chen [48]. The delta of the three indexes are reported in Table 3. The hypothesis that gender moderates the relationships between depression and romantic styles is confirmed.

### 7.1. Men

**Figure 1:**
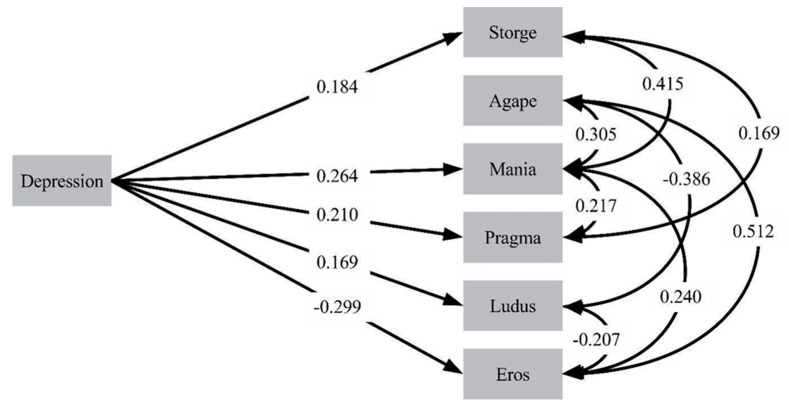
Final path analytic model of the effects of depression on attachment romantic styles in males.

Figure 1 shows the final path analytic model for male participants. In this model, depression was positively associated with mania, pragma, storge, and ludus, but negatively with eros. We did not have any significant relationships between depression and agape.

### 7.2. Women

**Figure 2:**
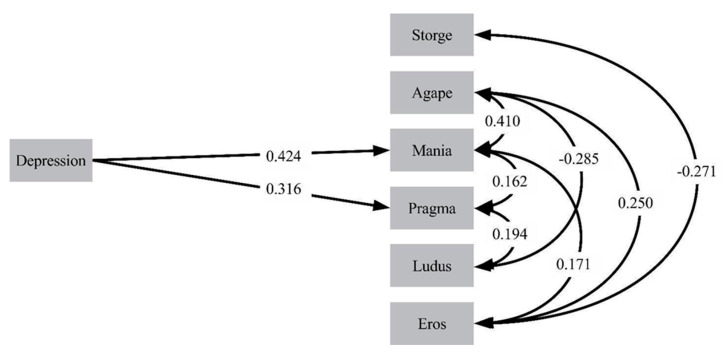
Final path analytic model of the effects of depression on attachment romantic styles in females.

Figure 2 shows the final path analytic model for female participants. In this model, depression was positively associated only with mania and pragma.

## 8. Conclusions

On the basis of the alarming data provide by ISTAT [5], according to which the percentage of Italian women who suffer from (psychological and/or physical) violence is increasing, we think it is important to identify the possible antecedent risk factors of such violence and act to oppose them.

The main goals of the present study were:

1. To verify whether depression has an impact on the development of unhealthy relationships and therefore, consequently, if it can play a role as a possible antecedent of aggression and victimization in romantic relationships;
2. To determine whether the gender variable moderates the relationship between depression and romantic styles of love in emerging adults.

In order to reach these aims, trying to fulfill the literature gaps, due to the fact that (1) depression is often considered as an outcome of unhealthy relationships and only rarely also as one of their antecedents [6], that (2) gender differences have not been adequately studied because all-women samples are generally the norm in research on romantic relationships and depressive symptoms [22], a self-report questionnaire was administered to evaluate the relation between depression symptoms and romantic style of love to a homogenous sample of 283 Italian emerging adults, consisting of 139 women and 144 men.

Data were analyzed by using the multigroup structural equation model. The model confirmed our hypotheses revealing that depression is a significant predictor for choosing and becoming involved in “unhealthy” love styles, and that gender moderates this relationship (i.e., the relationship between depression and love styles).

A multi-group structural equation model was also performed to test separate models revealing significant differences between men and women. Specifically, as far as men are concerned, the analysis showed strong associations between depression and pathological forms of love. In particular, although usually men have been found to be more erotic than women [49], a negative association between depression and eros has been identified, suggesting that men with higher levels of depression are less likely to be able to be engaged with or involved in a romantic or passionate love. According to our data, male depression is therefore a predictor of failure in establishing and maintaining healthy romantic forms of love. This result seems to be consistent with literature findings according to which “people with depressive symptoms struggle with impaired social skills […], diminished sexual interest […], questions about their relationship […], and reduced emotional disclosure” [50] (pp. 422–423).

Vice versa, positive associations have been found between depression and ludus, i.e., game-playing love. In other terms, depression in men seems to function by strengthening their tendencies to be more game-playing, more liberal, and less commitment-oriented in their behaviors than women, i.e., by boosting what is considered to be a typically masculine trait [49]. Nonetheless, positive associations have also been found between male depression on the one hand and storge (i.e., friendship love), pragma (i.e., logical and practical love), and mania (i.e., with possessive, dependent love) on the other, which are instead traditionally and socially recognized as (stereo)typically feminine ways of being engaged in love relations: “data indicated that females were more storgic (love as friendship) and pragmatic in their conception of love than males. There was also a tendency for females to be more manic” [49] (*p*. 184). Thus, depression seems to function also by shifting stereotypically masculine attitudes towards stereotypically feminine ones. Depression in men is therefore simultaneously a predictor not only for superficial (i.e., ludic) forms of love but also for friendly, practical and possessive ones. It is probable that these relationships comply with their needs of security and reassurance, which they are unable to find in themselves due to low self-esteem, which is a typical trait of depression [51]. Consistently with Sharabi et al. [50] (*p*. 434), depression is often characterized by dependence on relationships: “This dependence required the autonomous partner to devote extra effort to providing care, maintaining the relationship, and running the household”. No association was found between depression and agape (altruistic form of love), i.e., depression does not affect agape style of love. This means that agape is independent of men’s levels of depression.

In summary, depression in men is a predictor of manic (i.e., obsessive), ludic (i.e., superficial), friendly, or practical relationships. Men with high levels of depression do not seem to be able to establish relationships based on commitment, as required by the eros style.

Specifically, as far as women are concerned, unlike what happens for men, our analysis revealed that depression was positively associated exclusively with mania (i.e., with possessive, dependent love) and pragma (i.e., with logical and practical love), which are two of the traits identified as typically feminine ways to conceive love (e.g., [27,49,52]). In other words, depression in women seemed to function by reinforcing such traits (as observed with ludus for men). Therefore, women with high levels of depression were more frequently involved in, on the one hand, possessive and demanding relationships and, on the other, in practical ones (also similarly to what happens for men), confirming, as in the case of men, their need for dependence. Nonetheless, unlike what we observed for the men, depression in women seemed to be neither positively associated with other forms of unhealthy romantic styles nor negatively associated with healthy romantic ones.

Both for men and for women, our data revealed an increase in pragma and mania as a consequence of a high level of depression, i.e., depression was a significant predictor for choosing pragmatic and manic forms of love (independent of gender), in which the subjects, due to low levels of self-esteem, placed themselves in practical (pragmatic) and emotional (manic) dependence on a partner. Obviously, dependence can turn into possession, i.e., it can manifest itself through pathological, possessive behaviors in which a partner becomes dangerous to the subject of an obsession over control or attention, linked to the fear of losing him or her (“When my love affairs break up, I get so depressed that I have even thought of suicide” [27]).

We think that the identification of these risk factors can be useful to prevent intimate partner violence, specifically if they are used in order to prefigure and structure interventions of de-categorizations, which, according to Di Napoli et al. [53], should act at individual, relational, organizational, and community levels.

The implementation of these actions seems to be, in the Italian context, particularly urgent in the light of the alarming ISTAT [5] and Telefono Rosa [17] data.

## 9. Limitations

There are four main limitations to the present study. The first one concerns our sample. Although it was homogenous for gender and allowed us to fulfil the above-mentioned literature gap, it was numerically limited. Therefore, the results we obtained need further confirmations. For this reason, we think that for future research into this area it could be important to recruit a bigger and more representative sample.

Secondly, we did not take into consideration the role of parents’ attachment as a variable able to mitigate or moderate the effects of depressive symptoms on the choice of healthy romantic relationships. Accordingly with the literature on the topic, the “secure base script knowledge is positively associated with adults’ functioning in romantic relationships” although “the precise ways the attachment representations may influence adults’ thoughts, feelings and behaviors in close relationships remains limited” [21] (*p*. 2378). Feeney and Noller [54] identified a strong relationship between attachment styles and adult love relationships. According to their results, while secure subjects were trusting in their relationships and high in self-confidence, those with the avoidance style escaped intimacy and obtained high scores on ludus and low scores on the loving and romantic love, and those with the anxious-ambivalent style were characterized by dependence, desire for commitment in relationship, and involvement in neurotic forms of love. On the contrary, Hazan and Shaver [55] (pp. 522–523), although finding a certain continuity in attachment style between childhood and adulthood, obtained significant but not strong correlations: “The average person participates in several friendships and love relationships, each of which provides an opportunity to revise mental models of self and others”. The “attachment theory includes the idea that social development involves the continual construction, revision, integration, and abstraction of mental models […]. It would not be surprising to find that adult love is more complex than infant-caretaker attachment, despite fundamental similarities”. Analogously, Cassidy [56] (*p*. 126) also revealed that “some people are more likely to be continually influenced by early experiences, and some are more likely to be open to change. These variations contribute to the rich diversity of developmental trajectories”. Later attachment relationships can indeed be influenced, according to her, not only by children’s attachment to parents, but also by:

Relationships with other family members, which may be important in order to learn skills relevant to romantic relationships,

- observation of their parents’ romantic relationships,
- experiences with peers, and
- experiences in adulthood.

“Not only can new relationships change working models of the self, but situations and events outside of relationships can contribute to change as well”.

The third limitation concerns the direction of relationships we identified between depression and love styles. Although we assumed that high levels of depression (being stable predispositions) may increase the risk of engaging in unhealthy romantic styles, especially for men, the opposite assumption could also be true: that the unhealthy relationships might contribute to the development of depressive symptoms. The inability to be involved in healthy relationships can undermine self-esteem and cause a sense of inadequacy concerning the norms of traditionally masculine roles, according to which men have to maintain control in relationships. In other words, “men may experience greater anxiety because they may feel that they are not living up the social norms for masculinity” [11] (*p*. 1846). Our study furnishes a synchronic picture, but since it is not longitudinal research, it fails in distinguishing primary and secondary depression, i.e., it is unable to distinguish between depression as a cause and depression as an outcome.

Finally, the fourth limitation concerns the impact of cyberspace as a generator of changes in the aggressor-victim roles. As affirmed by Cuadrado-Gordillo and Fernández-Antelo [57] the increasing competence in using today’s communications and information technology that characterizes online society simplifies the posting and dissemination of anonymous messages. These characteristics of cyberspace can facilitate the emergence of cyber aggression [58].

Some of the main strengths of the present research consist of having shed light on a poorly investigated topic and in having underlined the role of gender as a variable that moderates the relationships between depression and romantic styles of love in emerging adults.

## Figures and Tables

**Table 1:** Regression analyses of the LAS (Love Attitudes Scale) on the CDI (Children’s Depression Inventory) as predictors in males.

LAS-CDI	Estimate	Std.Err	*z*-value	*p*(>|*z*|)	Std.lv	Std.all
Eros						
Depression	**−0.686**	**0.186**	**−3.692**	**0.000**	**−0.686**	**−0.299**
Ludus						
Depression	**0.393**	**0.194**	**2.026**	**0.043**	**0.393**	**0.169**
Storge						
Depression	**0.469**	**0.212**	**2.205**	**0.027**	**0.469**	**0.184**
Pragma						
Depression	**0.530**	**0.210**	**2.528**	**0.011**	**0.530**	**0.210**
Mania						
Depression	**0.593**	**0.184**	**3.225**	**0.001**	**0.593**	**0.264**
Agape						
Depression	0.028	0.194	0.144	0.885	0.028	0.012

Significant differences are in bold.

**Table 2:** Regression analyses of the LAS on the CDI as predictors in females.

LAS-CDI	Estimate	Std.Err	*z*-value	*p*(>|*z*|)	Std.lv	Std.all
Eros						
Depression	−0.201	0.198	−1.015	0.310	−0.201	−0.086
Ludus						
Depression	0.273	0.184	1.486	0.137	0.273	0.125
Storge						
Depression	0.011	0.236	0.048	0.962	0.011	0.004
Pragma						
Depression	**0.981**	**0.251**	**3.916**	**0.000**	**0.5981**	**0.316**
Mania						
Depression	**1.112**	**0.202**	**5.494**	**0.000**	**1.112**	**0.424**
Agape						
Depression	−0.093	0.220	−0.421	0.673	−0.093	−0.036

Significant differences are in bold.

**Table 3:** Results of invariance analyses across gender.

Invariance	ΔCFI	ΔRMSEA	ΔSRMR
Configural	0.000	0.000	0.000
Metric	0.010	0.011	0.016
Scalar	0.003	0.012	0.012

## References

[1] Fermani A., Bongelli R., Carrieri A., del Moral Arroyo G., Muzi M., Portelli C. (2019). “What is more important than love?” Parental attachment and romantic relationship in Italian emerging adulthood. Cogent Psychol..

[2] Attili G. (2017). Il Cervello in Amore.

[3] Hale W.W., Crocetti E., Raaijmakers Q.A., Meeus W.H. (2011). A meta-analysis of the cross-cultural psychometric properties of the Screen for Child Anxiety Related Emotional Disorders (SCARED). J. Child Psychol. Psychiatry.

[4] Sandberg D.A., Valdez C.E., Engle J.L., Menghrajani E. (2019). Attachment anxiety as a risk factor for subsequent intimate partner violence victimization: A 6-month prospective study among college women. J. Interpers. Violence.

[5] Italian National Institute of Statistics—ISTAT (2014). Popolazione. https://www.istat.it/.

[6] Yu R., Nevado-Holgado A.J., Molero Y., D’Onofrio B.M., Larsson H., Howard L.M., Fazel S. (2019). Mental disorders and intimate partner violence perpetrated by men towards women: A Swedish population-based longitudinal study. PLoS Med..

[7] Battisti A. (2019). I Numeri Della Violenza. https://www.slideshare.net/slideistat/a-battisti-i-numeri-della-violenza.

[8] Arnett J.J. (2000). Emerging adulthood: A theory of development from the late teens through the twenties. Am. Psychol..

[9] Godbout N., Daspe M.È., Runtz M., Cyr G., Briere J. (2019). Childhood maltreatment, attachment, and borderline personality–related symptoms: Gender-specific structural equation models. Psychol. Trauma.

[10] Rollè L., Giardina G., Caldarera A.M., Gerino E., Brustia P. (2018). When intimate partner violence meets same sex couples: A review of same sex intimate partner violence. Front. Psychol..

[11] Shorey R.C., Sherman A.E., Kivisto A.J., Elkins S.R., Rhatigan D.L., Moore T.M. (2011). Gender differences in depression and anxiety among victims of intimate partner violence: The moderating effect of shame proneness. J. Interpers. Violence.

[12] Dhariwal A., Connolly J., Paciello M., Caprara G.V. (2009). Adolescent peer relationships and emerging adult romantic styles: A longitudinal study of youth in an Italian community. J. Adolesc. Res..

[13] Pascuzzo K., Cyr C., Moss E. (2013). Longitudinal association between adolescent attachment, adult romantic attachment, and emotion regulation strategies. Attach. Hum. Dev..

[14] Lin K., Sun I.Y., Wu Y., Liu J. (2016). College students’ attitudes toward intimate partner violence: A comparative study of China and the US. J. Fam. Violence.

[15] Jiao Y., Sun I.Y., Farmer A.K., Lin K. (2016). College students’ definitions of intimate partner violence: A comparative study of three Chinese societies. J. Interpers. Violence.

[16] Sylaska K.M., Walters A.S. (2014). Testing the extent of the gender trap: College students’ perceptions of and reactions to intimate partner violence. Sex Roles.

[17] (2019). TELEFONO ROSA. http://telefonorosa.altervista.org/pdf.

[18] Camisasca E., Miragoli S., Di Blasio P., Grych J. (2017). Children’s coping strategies to inter-parental conflict: The moderating role of attachment. J. Child Fam. Stud..

[19] Signorelli M.S., Arcidiacono E., Musumesi G., Di Nuovo S., Aguglia E. (2014). Detecting domestic violence: Italian validation of revised Conflict Tactics Scale (CTS-2). J. Fam. Violence.

[20] Baldry A.C., Winkel F.W. (2008). Intimate Partner Violence Prevention and Intervention: The Risk Assessment and Management Approach.

[21] Waters T.E., Raby K.L., Ruiz S.K., Martin J., Roisman G.I. (2018). Adult attachment representations and the quality of romantic and parent–child relationships: An examination of the contributions of coherence of discourse and secure base script knowledge. Dev. Psychol..

[22] Anderson S.F., Salk R.H., Hyde J.S. (2015). Stress in romantic relationships and adolescent depressive symptoms: Influence of parental support. J. Fam. Psychol..

[23] Kamp Dush C.M., Arocho R., Mernitz S., Bartholomew K. (2018). The intergenerational transmission of partnering. PLoS ONE.

[24] Santona A., De Cesare P., Tognasso G., De Franceschi M., Sciandra A. (2019). The mediating role of romantic attachment in the relationship between attachment to parents and aggression. Front. Psychol..

[25] Crocetti E., Palmonari A., Palmonari A. (2011). Le fasi adolescenziali e giovanili nello sviluppo individuale [Adolescence and emerging adulthood as phases of the individual development]. Psicologia dell’Adolescenza [Psychology of Adolescence].

[26] Lee J.A. (1973). The Colors of Love: An Exploration of the Ways of Loving.

[27] Hendrick C., Hendrick S. (1986). A theory and method of love. J. Pers. Soc. Psychol..

[28] Crocetti E., Hale W.W., Fermani A., Raaijmakers Q., Meeus W. (2009). Psychometric properties of the Screen for Child Anxiety Related Emotional Disorders (SCARED) in the general Italian adolescent population: A validation and a comparison between Italy and The Netherlands. J. Anxiety Disord..

[29] Crick N.R., Nelson D.A., Morales J.R., Cullerton-Sen C., Casas J.F., Hickman S., Juvonen J., Graham S. (2001). Relational victimization in childhood and adolescence: I hurt you through the grapevine. Peer Harassment in School: The Plight of the Vulnerable and Victimized.

[30] Linder J.R., Crick N.R., Collins W.A. (2002). Relational aggression and victimization in young adults’ romantic relationships: Associations with perceptions of parent, peer, and romantic relationship quality. Soc. Dev..

[31] Collins K.A., Cramer K.M., Singleton-Jackson J.A. (2005). Love styles and self-silencing in romantic relationships. Guid. Couns..

[32] Arnett J.J. (2001). Conceptions of the transition to adulthood: Perspectives from adolescence through midlife. J. Adult Dev..

[33] Arnett J.J., Žukauskienė R., Sugimura K. (2014). The new life stage of emerging adulthood at ages 18–29 years: Implications for mental health. Lancet Psychiatry.

[34] Saunders M., Lewis P., Thornhill A. (2012). Research Methods for Business Students.

[35] R Core Team (2019). R: A Language and Environment for Statistical Computing.

[36] Kovacs M. (1985). The Children’s Depression Inventory (CDI). Psychopharmacol. Bull..

[37] Fermani A., Muzi M., Crocetti E., Meeus W. (2016). I genitori sono importanti per la chiarezza del concetto di Sé? Studenti e lavoratori a confronto. Psicol. Clin. Dello Svilupp..

[38] Beck A.T., Ward C.H., Mendelson M., Mock J., Erbaugh J. (1961). An inventory for measuring depression. Arch. Gen. Psychiatry.

[39] Crocetti E., Schwartz S.J., Fermani A., Meeus W. (2010). The Utrecht-management of identity commitments scale (U-MICS). Eur. J. Psychol. Assess..

[40] Bowling A. (2005). Mode of questionnaire administration can have serious effects on data quality. J. Public Health.

[41] Ullman J.B., Bentler P.M., Reynolds W.M., Miller G.E., Weiner I.B. Structural equation modeling. Handbook of Psychology.

[42] Bollen K.A., Curran P.J. (2006). Latent Curve Models: A Structural Equation Perspective.

[43] Schermelleh-Engel K., Moosbrugger H., Müller H. (2003). Evaluating the fit of structural equation models: Tests of significance and descriptive goodness-of-fit measures. Methods Psychol. Res. Online.

[44] Kline R.B. (2005). Principles and Practice of Structural Equation Modeling.

[45] Joreskog K.G., Sorbom D. (1996). LISREL8: User’s Reference Guide.

[46] Hooper D., Coughlan J., Mullen M.R. (2008). Structural equation modelling: Guidelines for determining model fit. Electron. J. Bus. Res. Methods.

[47] Ziegler M., Bensch D. (2013). Lost in translation: Thoughts regarding the translation of existing psychological measures into other languages. Eur. J. Psychol. Assess..

[48] Chen F.F. (2007). Sensitivity of goodness of fit indexes to lack of measurement invariance. Struct. Equ. Model. A Multidiscip. J..

[49] Hendrick C., Hendrick S., Foote F.H., Slapion-Foote M.J. (1984). Do men and women love differently?. J. Soc. Pers. Relat..

[50] Sharabi L.L., Delaney A.L., Knobloch L.K. (2016). In their own words: How clinical depression affects romantic relationships. J. Soc. Pers. Relat..

[51] Gabbard G.O. (2007). Psichiatria Psicodinamica.

[52] Smith R., Klases A. (2016). Predictors of love attitudes: The contribution of cultural orientation, gender attachment style, relationship length and age in participants from the UK and Hong Kong. Interpers. Int. J. Pers. Relatsh..

[53] Di Napoli I., Procentese F., Carnevale S., Esposito C., Arcidiacono C. (2019). Ending intimate partner violence (IPV) and locating men at stake: An ecological approach. Int. J. Environ. Res. Public Health.

[54] Feeney J.A., Noller P. (1990). Attachment style as a predictor of adult romantic relationships. J. Pers. Soc. Psychol..

[55] Hazan C., Shaver P.R. (1987). Romantic love conceptualized as an attachment process. J. Pers. Soc. Psychol..

[56] Cassidy J. (2000). Adult romantic attachments: A developmental perspective on individual differences. Rev. Gen. Psychol..

[57] Cuadrado-Gordillo I., Fernàndez-Antelo I. (2014). Cyberspace as a generator of changes in the aggressive-victim role. Comput. Hum. Behav..

[58] Cava M.J., Buelga S., Carrascosa L., Ortega-Baròn J. (2020). Relations among romantic myths, offline dating violence victimization and cyber dating violence victimization in adolescents. Int. J. Environ. Res. Public Health.

